# Klippel–Trenaunay and Sturge–Weber Overlap Syndrome with KRAS and GNAQ mutations

**DOI:** 10.1002/acn3.51106

**Published:** 2020-07-02

**Authors:** Ruojie He, Songjie Liao, Xiaoli Yao, Ruxun Huang, Jinsheng Zeng, Jian Zhang, Jian Yu

**Affiliations:** ^1^ Department of Neurology The First Affiliated Hospital Sun Yat‐sen University Guangdong Provincial Key Laboratory of Diagnosis and Treatment of Major Neurological Diseases National Key Clinical Department and Key Discipline of Neurology No.58 Zhongshan Road 2 Guangzhou 510080 China

## Abstract

Patients with combined phenotypes of Sturge–Weber syndrome and Klippel–Trenaunay syndrome have been reported, though the underlying genetic spectrum in these individuals remains to be elucidated. We reported the patient presenting with Klippel–Trenaunay and Sturge–Weber overlap syndrome in mainland China. Histopathologic study confirmed the hemangioma of vein and capillary. Co‐existence of a novel somatic *KRAS* c.182_183 delins TC mutation and *GNAQ* c.548G>A mutation was identified in the affected skin tissue rather than paired peripheral blood. The somatic mutations of *GNAQ* and *KRAS* may affect MAPK‐ERK signaling pathway, resulting in endothelial anomaly and blood vessel malformation.

## Introduction

Sturge–Weber syndrome is a sporadic congenital neurocutaneous disorder correlating to a somatic activating mutation in *GNAQ* c.548G>A (p.R183Q) by now, the hallmark of which is a facial port‐wine stain involving the ophthalmic trigeminal dermatome, leptomeningeal angioma, seizures, glaucoma, and intellectual disability.^1^ In contrast, Klippel–Trenaunay syndrome is a sporadic congenital vascular malformation with the most common somatic *PIK3CA* mutations as well as infrequent *KRAS* mutations, and distinguished by extensive port‐wine stains involving limbs and/or trunk associated with hypertrophy of soft tissue or bone and venous varicosities.^2^, ^3^ To date, Klippel–Trenaunay and Sturge–Weber overlap syndrome has been reported in several studies, where only the most common somatic *GNAQ* c.548G>A (p.R183Q) mutation without any other known mutations has been found.^4^, ^5^, ^6^ Nevertheless, whether a single somatic *GNAQ* mutation is responsible for the whole phenotypes remains to be determined.

Actually, two or more somatic gene mutations are likely to be involved in the Klippel–Trenaunay and Sturge–Weber overlap syndrome. Here, we reported a patient with Klippel–Trenaunay and Sturge–Weber overlap syndrome, and performed the Next Generation Sequencing (NGS) analysis of a panel of 524 common tumor genes to identify potential somatic mosaic mutations.

## Patient and Methods

### Subject

The study was conducted according to the Declaration of Helsinki and approved by the Institutional Review Board of The First Affiliated Hospital of Sun Yat‐sen University. Written informed consent was obtained from the patient. Clinical data including medical history, physical examination, cerebrospinal fluid (CSF) tests, computed tomography (CT), and magnetic resonance imaging scanning results from the patient were collected and analyzed.

### Histopathologic study

Five skin specimens with port‐wine stains from distinct affected the area including bilateral face, bilateral arms, and the middle of chest were obtained from the patient. All samples were fixed in 4% buffered formalin and embedded in paraffin. Tissue sections were cut and stained with hematoxylin and eosin.

### NGS study

Genomic DNA was purified from paired specimen of affected tissue from the right side of face and peripheral blood sample using the standard phenol extraction protocol. The NGS study was performed using the MSK‐IMPACT^™^ and FoundationOne CDx platform on an Illumina (San Diego, CA, USA) HiSeq 2000 sequencer to a mean per‐sample depth of coverage between 1700× and 2000×. The prospective cohort was analyzed using a panel targeting 524 cancer susceptibility genes including 93 hereditary cancer susceptibility genes (Table S1) (http://cancer.sanger.ac.uk/cosmic).

## Results

### Clinical findings

A 23‐year‐old Chinese man presented with recurrent seizures for more than 20 years. He was reported to develop epilepsy at the age of 1 year. When epilepsy happened, he suffered from squeezing headache on left temporal area, bilaterally blurred vision, and cramp of the limbs with loss of consciousness. After age of 10 years, the frequency of seizure gradually decreased. Around age of 16 years, the patient was blind in his left eye and the vision of right eye deteriorated progressively. At 10 days prior to hospitalization, the patient presented with manifestation of generalized tonic‐clonic seizure. When the seizure happened, he showed rigidity and cramp of four limbs with unconsciousness lasting 1–2 min, and reported eight episodes within 2 h. The patient could not provide medication history precisely. When he was hospitalized, he was administered with Levetiracetam (500 mg for once, twice per day) and had no seizure again. There were no varicosities on the extremities of the patient. At birth, the patient had extensive port‐wine stains on his body, but no extension in the past two decades. He had no other medical history. His mother's older brother had similar history of seizures, and his father had impaired vision.

**Figure 1:**
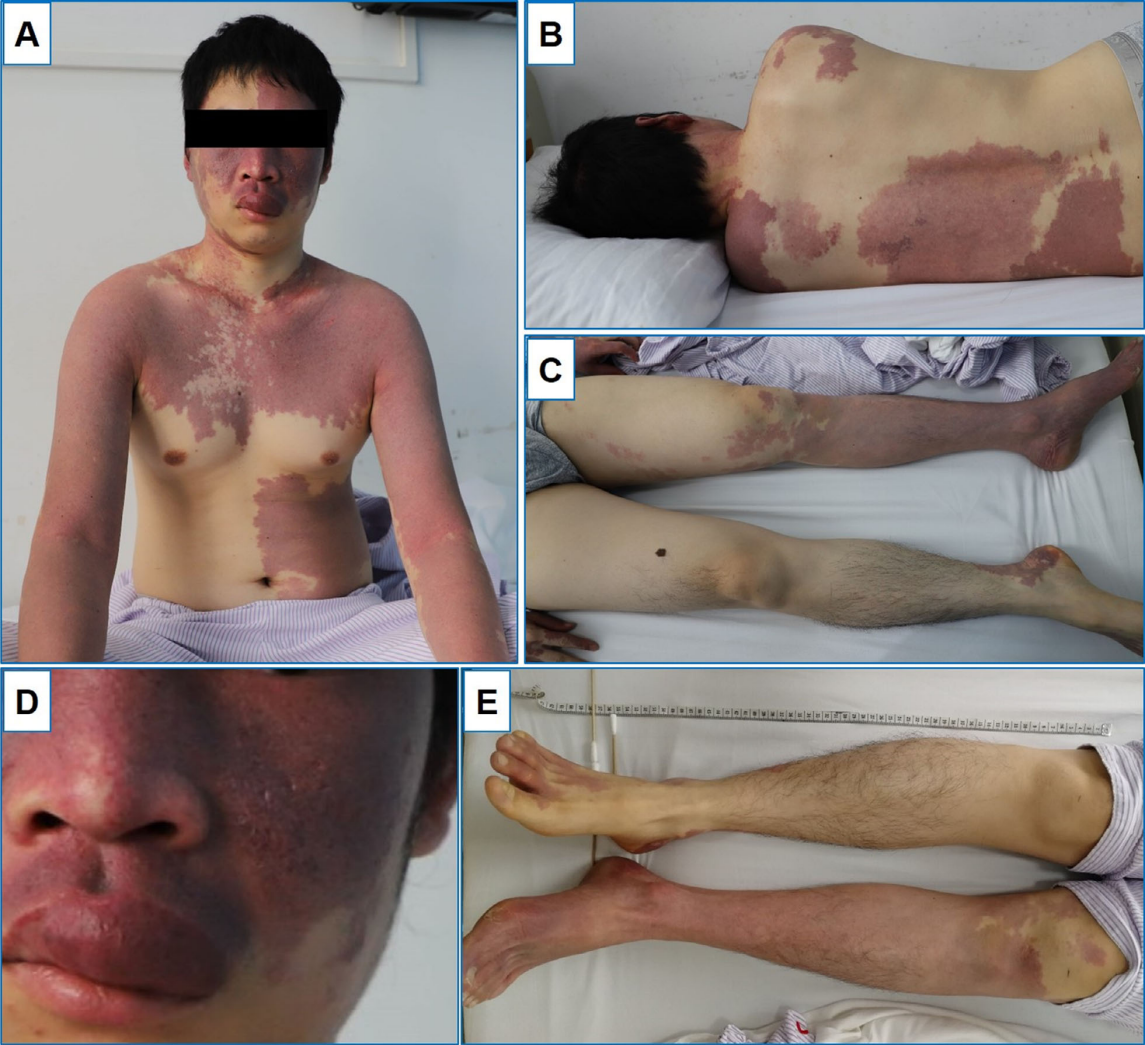
Photographs of the patient with Sturge–Weber syndrome and Klippel–Trenaunay syndrome. Photographs of the patient showed extensive port‐wine stains with involvement on his face, chest, abdomen, back and extremities (A–C), and the hypertrophy of upper lip region (D). The length of left knee to ankle was 57 cm and contralateral one was 55 cm, indicating the overgrowth of left lower extremity (E).

On general medical examination, this patient had extensive port‐wine stains involving his face, chest, abdomen, and extremities, asymmetric and more prominent on the left side of face and body (Fig. 1A–C). There was a melanocytic nevus on the right thigh. The hypertrophy of upper lip region (Fig. 1D) and overgrowth of left lower extremity were also observed (the length of left knee to ankle was 57 cm and contralateral one was 55 cm) (Fig. 1E). He had mild intellectual disability with a Wechsler Intelligence Scale score of 60. On neurological examination, his left eye was blind and there was just light sensation on the right eye, diagnosed as congenital cataract of the left eye and elevated intraocular pressure on the right eye. There was gaze‐evoked nystagmus in the horizontal gazes on the left eye, whereas the eye movements were normal. The power of upper and lower extremities was 5/5. Sensory examination showed that both vibratory sense and pinprick sense were diminished at the right side of trunk and extremities. There was hyperreflexia on four extremities, and bilateral Rossolimo's sign as well as Babinski's sign were positive.

**Figure 2:**
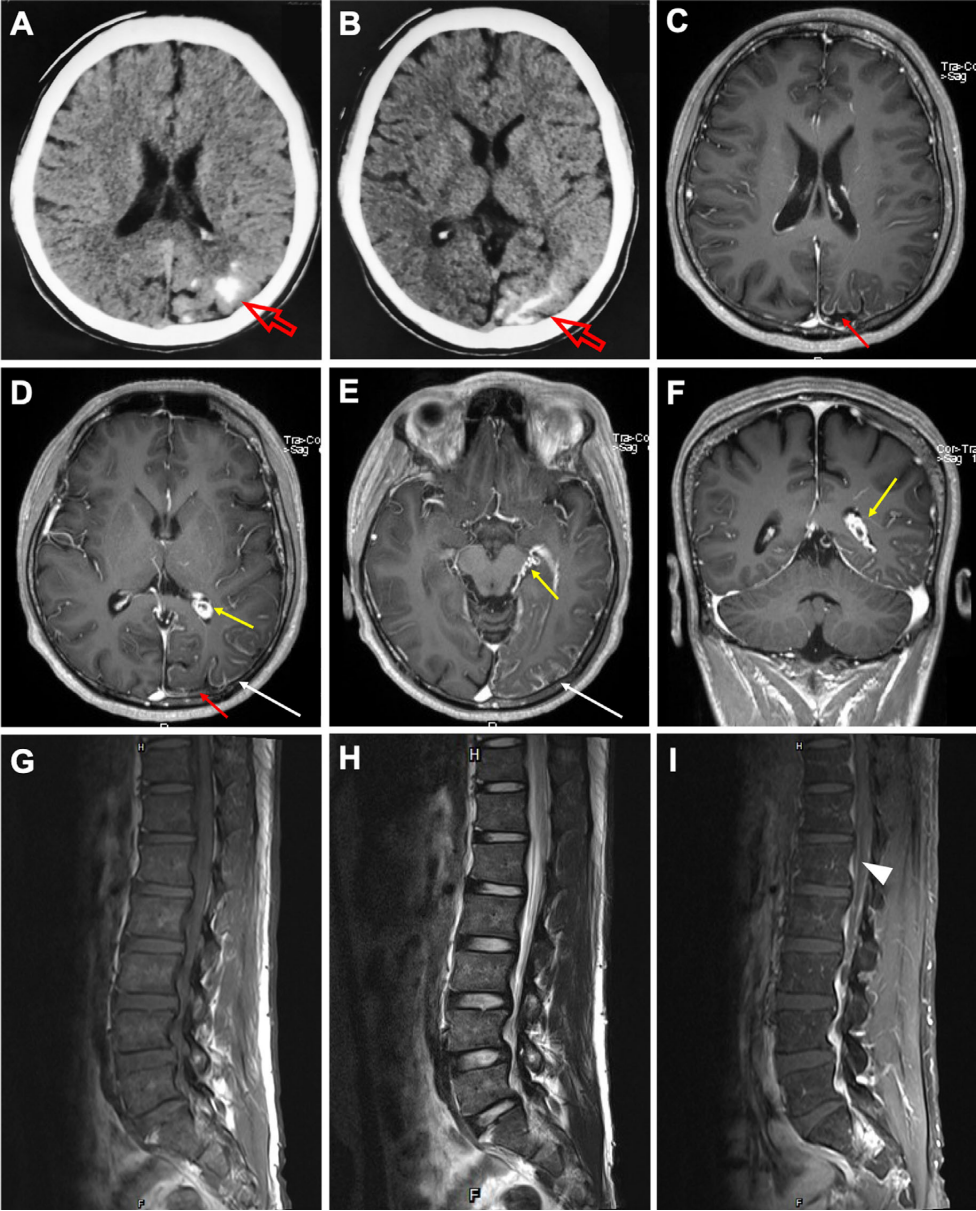
Representative images of brain from the patient with Sturge–Weber syndrome and Klippel–Trenaunay syndrome. Computed tomography scan showed the calcification in the left occipital lobe of the patient (open red arrows) (A, B). T1‐weighted post‐contrast magnetic resonance imaging (MRI) axial view showed left‐sided hemispheric leptomeningeal enhancement (white arrows), left hemispheric brain atrophy predominantly involving left occipital lobe (red arrows), and an enlarged and enhancing left‐sided choroid plexus (yellow arrows) (C–E). T1‐weighted post‐contrast MRI coronal view showed left‐sided choroid plexus enhancement (F). T1‐weighted and T2‐weighted MRI showed lumbosacral segments of spinal cord (G, H). T1‐weighted post‐contrast MRI showed the incrassation and significant enhancement of spinal meninges of lumbosacral segments (I) (arrowhead).

CT scan of head on admission revealed calcification in the left occipital lobe (Fig. 2A and B). MRI scan of brain with contrast showed significant enhancement of leptomeninges on left temporal lobe, parietal lobe, and occipital lobe with atrophy of cortex, as well as presentation of enhancing left‐sided choroid plexus adjoining left midbrain (Fig. 2C–F). Moreover, MRI scan of spinal cord with contrast revealed the incrassation and significant enhancement of spinal meninges including cervical, thoracic, and lumbosacral segments (Fig. 2G–I). Vascular ultrasound revealed malformation of left superficial femoral vein associated with enlarged left perforating vein and great saphenous vein. CSF analysis revealed mildly elevated protein level (1070.3 mg/L) with normal pressure, glucose level, and cell counts. Because the patient typically presented with extensive port‐wine stains, leptomeninges and choroidal hemangioma, ophthalmic involvement, recurrent seizures, as well as unilateral venous anomaly and enlargement of one limb, he was clinically suspected of having the Klippel–Trenaunay and Sturge–Weber overlap syndrome, and underwent genetic and pathological analyses.

### Histological and genetic findings

**Figure 3:**
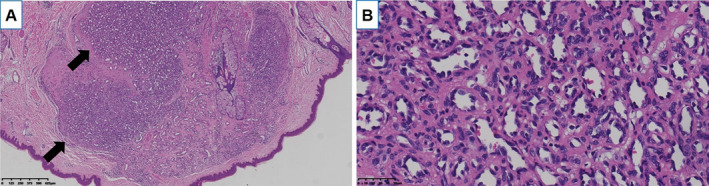
Histopathologic study of affected tissues from the patient. Hematoxylin and eosin (HE) staining on affected skin tissues demonstrated hyperplasia of small vessels in the dermis (A, black arrow, original magnification 40×), and the ectatic capillaries and small veins (B, original magnification 400×).

**Figure 4:**
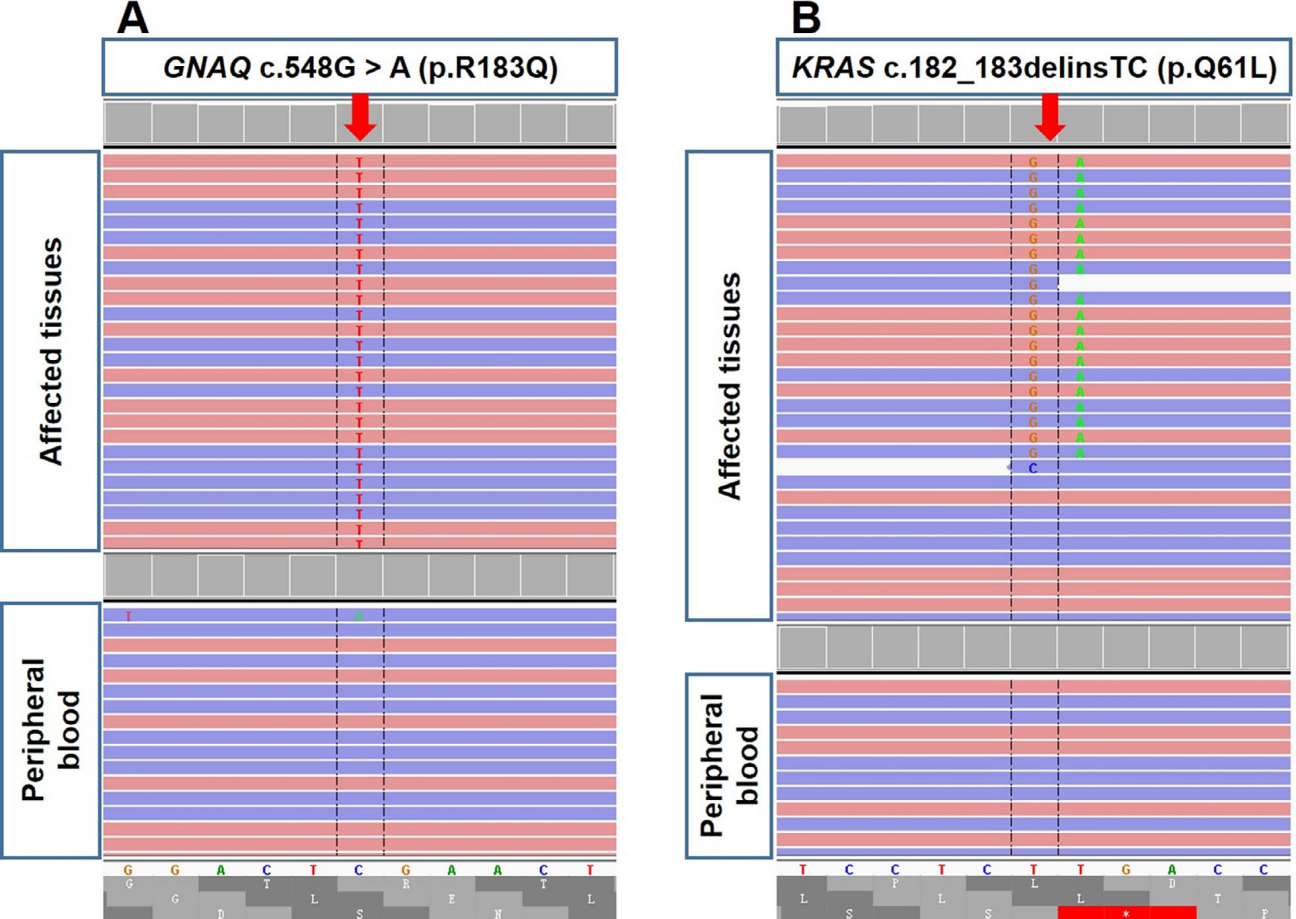
The Integrative Genomics Viewer (IGV) snapshot of Next generation sequencing (NGS) analysis identified somatic *GNAQ* and *KRAS* gene mutations in the affected tissues from the patient. NGS study demonstrated a *GNAQ* c.548G>A (p.R183KQ) at allele frequency of 11.72% in affected tissues; The C>T change (closed red arrow) on complementary strand was visualized by IGV snapshot in affected tissues, whereas no C>T change was identified in peripheral blood sample (A). NGS study demonstrated a *KRAS* c.182_183delinsTC (p.Q61L) at allele frequency of 3.54% in affected tissues; The deletion of TT and replacement with GA change (closed red arrow) on complementary strand was visualized by IGV snapshot in affected tissues, whereas no replacement of TT with GA was identified in peripheral blood sample (B). Each red and blue bar represents a sequencing read with base pairs matching the reference genome (The red one is forward sequencing read and the blue one is reverse sequencing read). Base cells deviating from the reference genome are considered as variants and are labeled.

Pathological detection of all affected skin tissues revealed hyperplasia and enlargement of small veins in the corium layer, confirming the diagnosis of hemangiomatosis (Fig. 3). In the NGS analysis, comparison of DNA sequences derived from affected tissue and paired peripheral blood sample revealed combined somatic mutations in *GNAQ* (NM_002072: c.548G>A (p.R183Q)) (Fig. 4A) and *KRAS* (NM_033360: c.182_183delinsTC (p.Q61L)) (Fig. 4B) with allele frequencies 11.72% and 3.54%, respectively. The *GNAQ* and the novel *KRAS* mutation were the only two mutations detected in the affected specimen from right side of face but not in the peripheral blood sample. Both of the mutations were activating in genes.

## Discussion

Sturge–Weber syndrome and Klippel–Trenaunay syndrome are two different entities within the spectrum of vascular malformations, predominantly caused by distinct somatic gene activating mutations. Here we reported the first pathologically and genetically confirmed case with combined phenotypes of Sturge–Weber syndrome and Klippel–Trenaunay syndrome. Clinically, the patient presented with recurrent seizures, intellectual disability, congenital cataract, facial port‐wine stain, and ipsilateral leptomeninges angioma, which were highly compatible with Sturge–Weber syndrome. In parallel, the extensive port‐wine stains involving his back, trunk, and extremities, unilateral venous anomaly, and enlargement of left lower extremity were also consistent with phenotypes of Klippel–Trenaunay syndrome. The further NGS analysis revealed a reported *GNAQ* somatic variation and a novel *KRAS* somatic mutation in the patient.

Previously, an activating somatic c.548G>A (p.R183Q) mutation in the *GNAQ* gene, the first identified and the most common pathogenic substitution, has been found in association with both Sturge–Weber syndrome and non‐syndromic facial port‐wine stains.^1^, ^7^, ^8^ *GNAQ* encodes guanine nucleotide‐binding protein (G protein) that contributes to hydrolyze the intracellular messenger GTP to GDP, a key step required for inactivation of the protein.^1^ *GNAQ* p.Arg183Gln substitution may reduce the GTPase activity, leading to increased signaling of MAPK‐ERK.^1^ Indeed, further studies have also suggested that activating *GNAQ* mutations are likely to induce changes in cellular morphology and cell growth mainly via upregulating the MAPK‐ERK signaling pathway, providing a foundation of molecular pathogenesis on Sturge–Weber syndrome.^9^, ^10^ The *KRAS* gene, a member of the RAS oncogene family, encodes a protein that is a member of the small GTPase superfamily with function of transducing extracellular signals to intracellular signal cascades. *KRAS* activating mutations have been found to specifically activate the MAPK‐ERK signaling pathway in endothelial cells derived from arteriovenous malformations of the brain, indicated by the increased level of ERK1/2 phosphorylation in endothelial cells cultures with mutant *KRAS*.^11^ Moreover, a study from Al‐Olabi, et al. found that multiple mosaic‐activating variants in four genes including *KRAS* associated with the activated MAPK pathway would cause sporadic vascular malformations.^12^ Intriguingly, a recent study revealed that several somatic *KRAS* mutations like *KRAS* c.64C>A (p.Gln22Lys) and c.436G>A (p.Ala146Thr) were associated with combined phenotypes of Klippel–Trenaunay syndrome and port‐wine stains.^2^ In this study, we found a novel *KRAS* c.182_183delinsTC (p.Q61L) mutation in the patient with Klippel–Trenaunay syndrome.

To our knowledge, such a combination of somatic *GNAQ* and *KRAS* mutations in vascular malformations or overgrowth syndromes has not been reported before. The somatic *GNAQ* c.548G>A mutation detected in our patient is a typical pathogenic variation that has been reported in previous studies. In contrast, the novel somatic *KRAS* c.182_183delinsTC mutation is identified for the first time in the present study. This variation results in an amino acid substitution of Glutamine into Leucine. From a signaling transduction perspective, we speculate that the pathogenic mutations in *GNAQ* and *KRAS* may provide compatible function in parallel with MAPK‐ERK signaling pathway activation. Further studies should be performed to elucidate the pathogenic mechanism of *GNAQ* and *KRAS* activating mutations in the development of Sturge–Weber syndrome and Klippel–Trenaunay syndrome.

In conclusion, we reported a patient presenting with the combination phenotypes of Sturge–Weber syndrome and Klippel–Trenaunay syndrome, and identified the co‐occurrence of somatic mosaic *GNAQ* and *KRAS* mutations for the first time using the NGS analysis. Our results demonstrated that in patients with the overlapping phenotypes of Sturge–Weber syndrome and Klippel–Trenaunay syndrome, both *GNAQ* and *KRAS* gene should be tested to identify the pathogenic somatic mutations.

## Author Contribution

RJH conducted the clinical investigations and wrote the manuscript. SJL, XLY, RXH, and JSZ conceptualized the case. JZ interpreted the data. JY conceived the study and revised the manuscript.

## Conflict of Interest

The authors declare no conflicts of interest.

## Supporting information


**Table S1.** The panel of 524 cancer susceptibility genes including 93 hereditary cancer susceptibility genes targeted in next generation sequencing (NGS) analysis in present study.
